# Serum insulin-like growth factor-1 is lower in children with type 1 and type 2 diabetes mellitus than in a non-diabetic comparison group after adjustment for age and sex: a cross-sectional study

**DOI:** 10.3389/fped.2026.1948533

**Published:** 2026-10-13

**Authors:** Yuxia Zhang, Dingding Wang, Wei Song

**Affiliations:** Department of Pediatrics, The Second Qilu Hospital of Shandong University, Jinan, Shandong, China

**Keywords:** C-peptide, growth hormone, insulin-like growth factor 1, type 1 diabetes mellitus, type 2 diabetes mellitus

## Abstract

**Introduction:**

Few studies have compared insulin-like growth factor-1 (IGF-1) in children with type 1 (T1DM) and type 2 diabetes (T2DM) against a shared non-diabetic reference group.

**Methods:**

We retrospectively analyzed data from 70 children with T1DM, 14 with T2DM, and 196 children in a non-diabetic comparison group. IGF-1, HbA1c, C-peptide, and anthropometric data were compared using nonparametric tests and multivariable regression of log-transformed IGF-1 adjusted for age and sex, with age-adjusted partial correlations for C-peptide, HbA1c, and BMI. As a supportive analysis, IGF-1 in T1DM (*n* = 57) and T2DM (*n* = 13) was converted to age- and sex-specific SDS using an external reference and tested against zero.

**Results:**

Unadjusted IGF-1 was lowest in T1DM (145.0 ng/mL), intermediate in T2DM (232.0 ng/mL), and highest in the comparison group (276.5 ng/mL; *P* < 0.001), reflecting the younger age of the T1DM group. After adjustment, IGF-1 was lower in T1DM (−41.8%, *P* < 0.001) and T2DM (−39.4%, *P* = 0.003) than in the comparison group. No significant difference between T1DM and T2DM was detected (*P* = 0.83), but the 95% confidence interval was wide (−31.3% to +55.9%) because only 14 children had T2DM. Externally referenced SDS was significantly below zero in T1DM (*P* = 0.009) but not in T2DM (*P* = 0.33). C-peptide, near-absent in T1DM and preserved in T2DM, was not correlated with IGF-1 in either group.

**Discussion:**

These exploratory findings show lower age- and sex-adjusted IGF-1 in both diabetes subtypes, leave open whether the reduction differs between them, and warrant prospective studies including pubertal staging and GH-axis measurements.

## Highlights

- After adjustment for age and sex, IGF-1 was lower in children with T1DM and T2DM than in a non-diabetic comparison group.
- No significant difference between T1DM and T2DM was detected, but the small T2DM sample (*n* = 14) limits this comparison.
- IGF-1 was lower in both groups despite very different residual *β*-cell function.
- IGF-1 was the primary hormonal measure; thus, mechanistic insights remain hypothetical and require validation with direct GH-axis measurements.

## 1 Introduction

Diabetes mellitus in childhood encompasses at least two pathophysiologically distinct entities: type 1 diabetes mellitus (T1DM), driven by autoimmune *β*-cell destruction and near-total insulin deficiency, and type 2 diabetes mellitus (T2DM), characterized by insulin resistance with variably preserved endogenous insulin secretion. Both conditions arise during a period of life in which normal growth is critically dependent on an intact growth hormone (GH)/insulin-like growth factor-1 (IGF-1) axis.

Hepatic IGF-1 synthesis does not depend on GH alone; adequate portal insulin exposure is also necessary to sustain hepatocyte GH receptor expression and hepatic GH sensitivity. When portal insulin delivery is chronically insufficient, as occurs physiologically during fasting and pathologically in T1DM (where subcutaneous insulin administration fails to reproduce normal portal insulin dynamics), hepatic GH resistance develops. GH secretion then rises compensatorily, yet circulating IGF-1 remains low (1–3). This mechanism has been demonstrated in both clinical and rodent studies of insulin-deficient diabetes and has prompted investigation of GH-axis-targeted adjunctive therapies for T1DM (4).

Lower serum IGF-1 has been consistently reported in children and adolescents with T1DM compared with non-diabetic comparison groups (5–7). This deficit tends to become more pronounced with poorer glycemic control and longer disease duration (8, 9), and it tracks with declining residual *β*-cell function (10, 11). It remains less clear whether IGF-1 is also reduced in pediatric T2DM, a condition in which portal insulin exposure is not deficient but insulin action is impaired by resistance, particularly since few studies have directly compared T2DM with T1DM using a shared non-diabetic reference group.

We used cross-sectional clinical data from a single pediatric center to (1) compare serum IGF-1 across children with T1DM, T2DM, and a non-diabetic comparison group, explicitly accounting for the age dependence of IGF-1; (2) examine whether IGF-1 correlates with residual *β*-cell function (C-peptide) and glycemic control (HbA1c) within each diabetes subtype.

## 2 Materials and methods

### 2.1 Study design and data sources

This was a retrospective, single-center, cross-sectional analysis of clinical laboratory data collected during routine care at the Department of Pediatrics. Patient records from 2018 to 2024 were retrospectively reviewed. Two institutional datasets were integrated: (i) a non-diabetic comparison group of 196 children; (ii) a cohort of 70 children with T1DM and 14 children with T2DM with concurrent IGF-1, C-peptide, HbA1c, and anthropometric data. The inclusion criteria for the T1DM group were age 3–14 years and meeting the diagnostic criteria for T1DM (12): (1) symptoms of diabetes (polyuria, polydipsia, unexplained weight loss) with a casual plasma glucose ≥200 mg/dL (11.1 mmol/L), casual being defined as any time of day without regard to time since the last meal; or (2) fasting plasma glucose ≥126 mg/dL (7.0 mmol/L); or (3) 2-hour plasma glucose ≥200 mg/dL (11.1 mmol/L) during an oral glucose tolerance test performed per World Health Organization criteria [75 g anhydrous glucose dissolved in water, or 1.75 g/kg body weight for children weighing <40 lb (18 kg)]; or (4) fasting basal plasma insulin ≤5 *μ*IU/mL together with fasting plasma glucose ≥126 mg/dL. Eligible T1DM patients had no signs or symptoms of diabetes complications, no acute illness within the 3 months preceding study entry, and were treated with a basal-bolus insulin regimen of multiple daily injections (MDI) or continuous subcutaneous insulin infusion (CSII). Diabetes type was assigned by the treating pediatric endocrinologist and documented in the clinical record, based on clinical presentation, fasting C-peptide and treatment course. The non-diabetic comparison group comprised children who attended routine check-ups at the child health-care outpatient clinic and whose height did not meet the criteria for short stature. An oral glucose tolerance test was not performed. Chronic disease and medication use were screened. Because glucose tolerance was not tested, we refer to them as a non-diabetic comparison group. Although islet autoantibody testing (GAD/IAA/ICA) was performed at our center, changes in reagents and analyzers across the study period (2018–2024) precluded data harmonization. Therefore, autoantibody results were not incorporated into the diagnostic criteria, which we note as a limitation. Fasting C-peptide and fasting glucose (Table 1) were obtained from the same blood draw at each visit, and assay methodology for both analytes was consistent throughout the 2018–2024 study period, providing a concurrent, internally consistent basis for interpreting residual *β*-cell secretory capacity relative to ambient glycemia. Serum IGF-1 was measured by chemiluminescence immunoassay (CLIA) with the YHLO IGF-1 kit (Shenzhen YHLO Biotech Co., Ltd.). According to the manufacturer, the kit is registered with the Guangdong provincial medical products administration, the calibrators are traceable to the WHO International Standard 19/166 (second-generation IGF-1 standard), and the lower limit of quantification is approximately 25 ng/mL. Age- and sex-specific reference intervals followed the Chinese group standard T/GDAQ 00009-2023.

**Table 1:** Baseline characteristics by group.

Characteristic	T1DM	T2DM	Comparison group	Overall P^a^	Post-hoc pairs^b^
n	70	14	196	—	—
Male, *n* (%)	41 (59.4)	12 (85.7)	114 (43.7)	—	—
Age, years	8.0 [4.0–10.0]	10.0 [8.0–13.8]	10.0 [8.0–12.0]	<0.001	T1 < T2*; T1 < Comparison***
BMI, kg/m^2^	16.8 [15.2–18.4]	22.1 [15.4–25.9]	17.4 [15.5–20.6]	0.133	NS
Fasting glucose, mmol/L	8.9 [5.4–11.7]	7.9 [7.1–11.2]	5.3 [5.0–5.6]	<0.001	T1,T2 > Comparison***
HbA1c, %	7.9 [7.0–9.8]	10.5 [6.3–12.3]	N/A	0.635^c^	T1≈T2
Fasting C-peptide, ng/mL	0.09 [0.00–0.32]	1.92 [1.19–2.54]	1.80 [1.31–2.18]	<0.001	T1 < T2,Comparison***
Serum IGF-1, ng/mL	145.0 [82.6–173.0]	232.0 [116.4–268.0]	276.5 [176.5–437.5]	<0.001	T1 < T2*; T1 < Comparison***

^a^*Kruskal–Wallis test across the three groups (or Mann–Whitney where only two groups had data).* ^b^Pairwise Mann–Whitney: *P *<* 0.05, **P *<* 0.01, ***P *<* *0.001; NS* *=* *not significant.* ^c^*Comparison limited to T1DM vs T2DM (comparison-group HbA1c not systematically available in this dataset).*

### 2.2 Statistical analysis

Continuous variables are reported as median [interquartile range] given non-normal distributions. Group comparisons used the Kruskal–Wallis test for the overall (three-group) comparison, followed by pairwise Mann–Whitney U tests. Because IGF-1 rises steeply and non-linearly across childhood and puberty, and the T1DM group was significantly younger than the comparison group, group differences in log-transformed IGF-1 were additionally examined using multivariable linear regression with age (years), sex, and diabetes group (T1DM, T2DM, comparison group as reference) as covariates; coefficients were back-transformed to percentage differences in IGF-1. A linear age term was retained in this primary model as a parsimonious specification given the modest subgroup sizes, particularly for T2DM (*n* = 14); the non-linear, pubertal shape of the age-IGF-1 relationship is only partly addressed by the complementary, LMS-based standard deviation score (SDS) analysis described in Section 3.3, which compares the diabetes groups with an external reference. Associations between IGF-1 and C-peptide, HbA1c, and BMI within each diabetes subtype were assessed using Spearman's rank correlation, and, given the strong confounding effect of age, also as partial (age-adjusted) Spearman correlations. As a supportive analysis that does not rely on the internal comparison group, IGF-1 values in the T1DM and T2DM groups were additionally converted to age- and sex-specific standard deviation scores (SDS) using the LMS method with published international reference L and M parameters (13) (chemiluminescence immunoassay reference spanning birth to senescence, *N* = 15,014; non-integer ages interpolated linearly between adjacent integer-age L/M values; constant S = 0.4568 for males and S = 0.4595 for females). This reference-based SDS was tested against a hypothesized reference-population median of 0 using one-sample Wilcoxon signed-rank tests within each diabetes subtype, and compared between T1DM and T2DM using the Mann–Whitney U test. Two-sided *P* < 0.05 was considered statistically significant; given the exploratory nature of this analysis, *P* values are reported without formal multiplicity correction and should be interpreted as hypothesis-generating.

### 2.3 Ethical considerations

This analysis used de-identified, aggregate laboratory and anthropometric data derived from existing clinical records. No patient names or other direct identifiers were used in any analysis or are reported in this manuscript. This study was approved by the Institutional Ethics Committee of the Second Qilu Hospital of Shandong University [IRB reference: KYLL-2018(KJ)P-0016], which waived the requirement for informed consent given the retrospective design. All patient data were de-identified in compliance with the Declaration of Helsinki.

## 3 Results

### 3.1 Baseline characteristics

Table 1 summarizes baseline characteristics of the three groups. The T1DM group was significantly younger than both the T2DM and comparison groups (*P* < 0.001), while T2DM and the comparison group did not differ significantly in age. Fasting glucose was markedly elevated in both diabetes groups compared with the comparison group, with no difference between T1DM and T2DM. Median HbA1c was 7.9% in T1DM and 10.5% in T2DM, without a significant difference (*P* = 0.635). Fasting C-peptide differed between the two diabetes subtypes, consistent with the classification criteria (which included C-peptide): T1DM patients had near-absent C-peptide (median 0.09 ng/mL), whereas T2DM patients had preserved C-peptide (median 1.92 ng/mL), not significantly different from the comparison group (median 1.80 ng/mL, *P* = 0.539).

### 3.2 Serum IGF-1 is reduced in both T1DM and T2DM

Unadjusted serum IGF-1 differed significantly across the three groups (Kruskal–Wallis *P* < 0.001; Figure 1), with the lowest median values in T1DM (145.0 ng/mL), intermediate values in T2DM (232.0 ng/mL), and the highest values in the comparison group (276.5 ng/mL). In pairwise comparisons, T1DM was significantly lower than both the comparison group (*P* < 0.001) and T2DM (*P* = 0.043); the T2DM-versus-comparison difference did not reach significance in the unadjusted analysis (*P* = 0.142).

**Figure 1:**
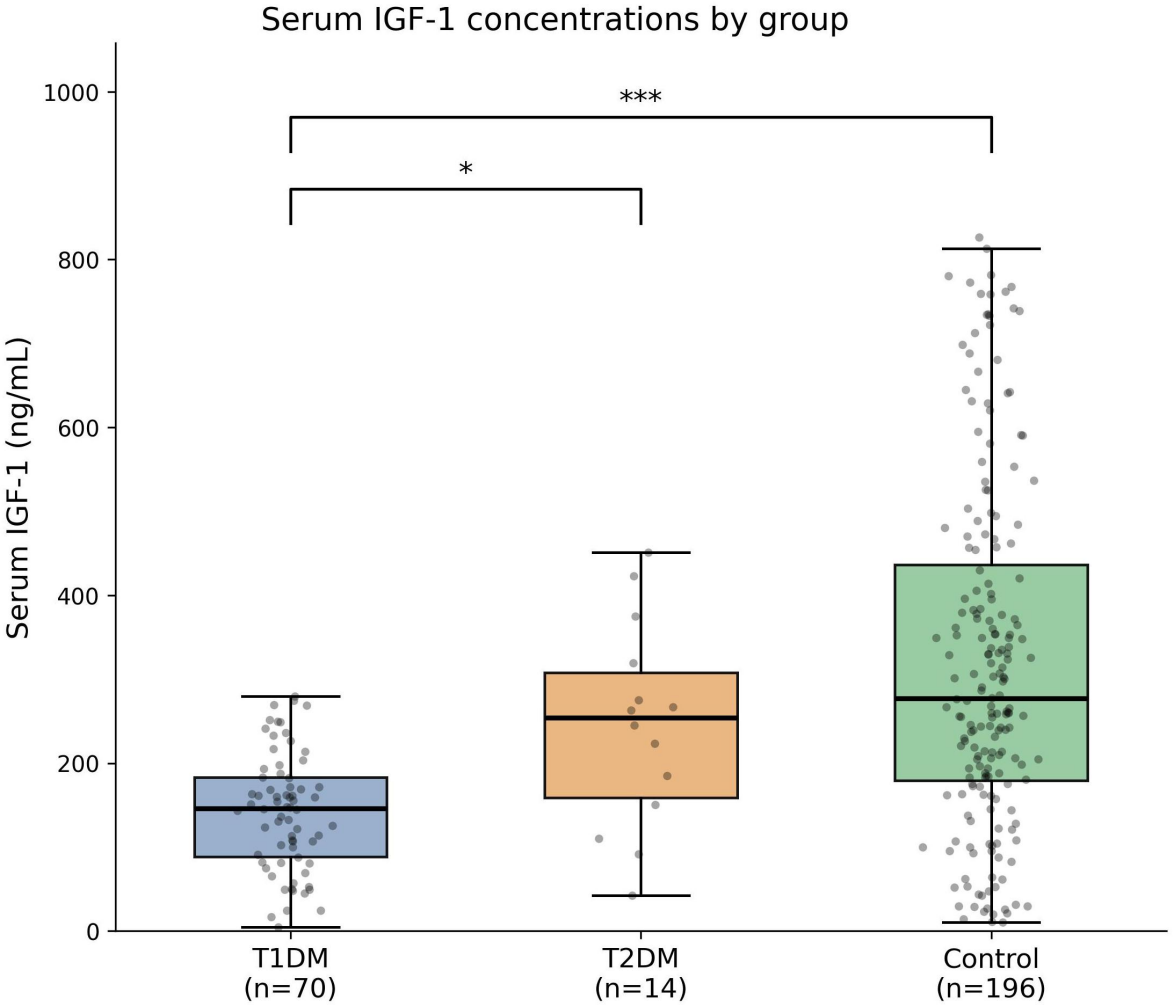
Serum IGF-1 concentrations by group. Boxes show median and interquartile range; points show individual values (outliers beyond the whiskers not separately plotted). Brackets denote unadjusted pairwise comparisons: ***P < 0.001 (T1DM vs comparison group); *P < 0.05 (T1DM vs T2DM); the T2DM-vs-comparison group comparison was not significant (*P* = 0.142).

IGF-1 correlated strongly and positively with age in every group (T1DM *ρ*=0.64; T2DM *ρ*=0.83; Comparison *ρ*=0.60; all *P* < 0.001; Table 2). A multivariable linear regression of log-transformed IGF-1 on age, sex, and diabetes group was fitted (Table 3). After adjustment, each additional year of age was associated with a 13.0% higher IGF-1 (95% CI 10.6-15.4%, *P* < 0.001), and female sex was associated with 28.1% higher IGF-1 than male sex (95% CI 9.6-49.7%, *P* = 0.002). Both T1DM (−41.8%, 95% CI −51.7 to −29.8%, *P* < 0.001) and T2DM (−39.4%, 95% CI −56.5 to −15.6%, *P* = 0.003) remained significantly lower than in the comparison group after this adjustment, and there was no significant difference between T1DM and T2DM once age and sex were accounted for (+4.0% for T2DM vs T1DM, 95% CI −31.3 to 55.9%, *P* = 0.829; an interval compatible with substantial differences in either direction, because only 14 children had T2DM). This age-adjusted model explained 45.3% of the variance in log-IGF-1 (R^2^ = 0.453, *n* = 259) (Figure 2).

**Table 2:** Spearman and age-adjusted partial correlations between IGF-1 and clinical variables, by group.

IGF-1 correlated with	T1DM *ρ* (P), n	T2DM *ρ* (P), n	Comparison group *ρ* (P), n
Age	0.64 (<0.001), 57	0.83 (<0.001), 14	0.60 (<0.001), 192
BMI	0.28 (0.035), 55	0.08 (0.79), 14	0.49 (<0.001), 94
HbA1c	−0.11 (0.41), 58	−0.29 (0.31), 14	N/A
Fasting C-peptide	0.12 (0.37), 58	−0.16 (0.59), 14	0.59 (<0.001), 187
C-peptide, age-adjusted^a^	0.17 (0.20), 57	−0.48 (0.10), 14	—
HbA1c, age-adjusted^a^	−0.19 (0.16), 57	−0.40 (0.18), 14	—

a *Partial Spearman correlation controlling for age (not calculable for the comparison group's HbA1c, which was not systematically available; C-peptide/BMI age-adjusted partials for the comparison group are omitted here as unadjusted associations were already highly significant and consistent in direction).*

**Table 3:** Multivariable linear regression of log-transformed IGF-1 on age, sex, and diabetes group.

Predictor	% difference in IGF-1	95% CI	*P* value
Age (per year)	+13.0%	10.6 to 15.4	<0.001
Female sex (vs male)	+28.1%	9.6 to 49.7	0.002
T1DM (vs comparison group)	−41.8%	−51.7 to −29.8	<0.001
T2DM (vs comparison group)	−39.4%	−56.5 to −15.6	0.003
T2DM (vs T1DM)^a^	+4.0%	−31.3 to 55.9	0.829

a *Model re-parameterized with T1DM as the reference category to directly test the T1DM-vs-T2DM contrast.*

**Figure 2:**
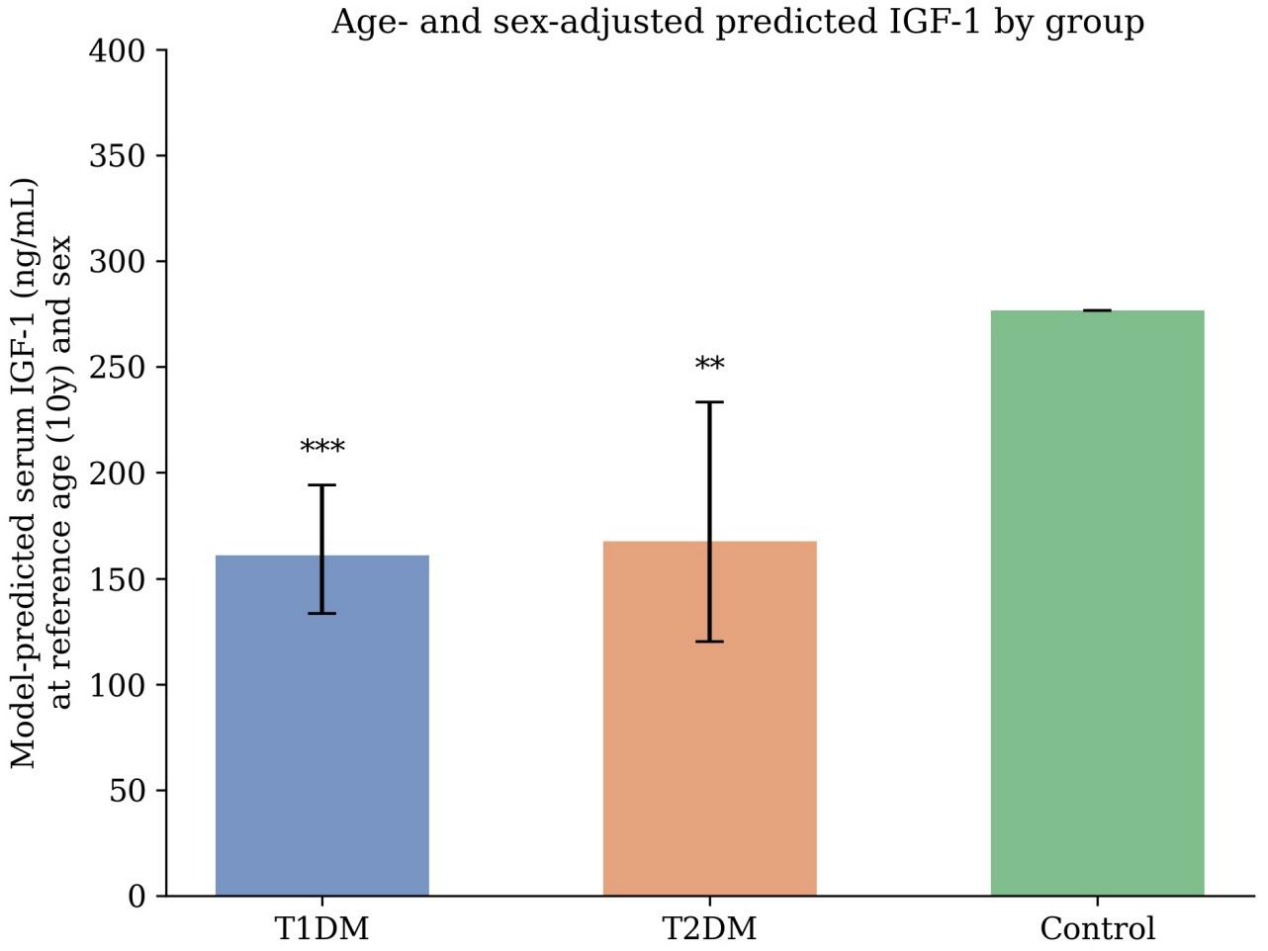
Age- and sex-adjusted predicted serum IGF-1 by group, derived from the multivariable regression model in Table 3. Bars show model-predicted IGF-1 at a common reference age (10 years) and reference sex, holding age and sex constant across groups; the comparison-group bar reflects the model-predicted value at the same reference age and sex used for T1DM and T2DM, and the T1DM and T2DM bars apply the adjusted percentage differences (with 95% CI) from Table 3 to that reference. Error bars show 95% CI. ***P < 0.001, **P < 0.01 versus the comparison group; NS, not significant (T1DM vs T2DM, *P* = 0.829).

### 3.3 Supportive analysis using an externally referenced IGF-1 SDS

To complement the age-adjusted comparison above using a standard independent of our institution's internal comparison group, IGF-1 values in T1DM (*n* = 57) and T2DM (*n* = 13) with complete age and sex data were converted to age- and sex-specific SDS using the published international LMS reference of Bidlingmaier et al. (13) (Supplementary Table S1). Median IGF-1 SDS was −0.32 (IQR −1.17 to 0.40) in T1DM, significantly below the reference population median of 0 (one-sample Wilcoxon *P* = 0.009; mean −0.58 ± 1.52), with 68.4% of patients falling below the reference median, 31.6% below −1 SDS, and 14.0% below −2 SDS. In T2DM, median IGF-1 SDS was −0.54 (IQR −1.14 to 0.49; mean −0.36 ± 1.42), numerically in the same direction as in T1DM (53.8% below the reference median, 30.8% below −1 SDS, 15.4% below −2 SDS), but this did not reach statistical significance against the reference population (*P* = 0.33). IGF-1 SDS did not differ significantly between T1DM and T2DM (Mann–Whitney *P* = 0.81). Within each subtype, IGF-1 SDS was not significantly associated with C-peptide or HbA1c (T1DM: *ρ*=0.12 and *ρ*=−0.21 respectively, both *P* > 0.10, *n* = 57; T2DM: *ρ*=−0.43 and *ρ*=−0.40 respectively, both *P* > 0.10, *n* = 13).

### 3.4 IGF-1 and residual *β*-cell function/glycemic control within diabetes subtypes

Within T1DM, IGF-1 correlated weakly with C-peptide (*ρ*=0.12, *P* = 0.37, *n* = 58) and HbA1c (*ρ*=−0.11, *P* = 0.41), neither significant. These associations remained non-significant after adjusting for age (partial *ρ*=0.17, *P* = 0.20 for C-peptide; partial *ρ*=−0.19, *P* = 0.16 for HbA1c). Within T2DM, IGF-1 showed a moderate negative age-adjusted partial correlation with both C-peptide (partial *ρ*=−0.48, *P* = 0.10, *n* = 14) and HbA1c (partial *ρ*=−0.40, *P* = 0.18), neither reaching statistical significance. In the comparison group, IGF-1 correlated positively with both BMI (*ρ*=0.49, *P* < 0.001) and C-peptide (*ρ*=0.59, *P* < 0.001). Full correlation results are summarized in Table 2.

## 4 Discussion

This exploratory cross-sectional analysis found lower serum IGF-1 in children with T1DM and in children with T2DM than in the non-diabetic comparison group after adjustment for age and sex, with no significant difference detected between the two subtypes; because only 14 children had T2DM, the study cannot establish whether the magnitude of the reduction differs between subtypes. This finding extends prior reports of reduced IGF-1 in pediatric T1DM by suggesting that IGF-1 is also lower in T2DM, despite the different insulin secretory status of the two conditions. In our cohort, T1DM patients exhibited near-total *β*-cell failure, whereas T2DM patients showed preserved C-peptide levels similar to those of the comparison group. The unadjusted difference in absolute IGF-1 between the subtypes likely reflects their differing age and pubertal distributions rather than a disease-specific effect. The T1DM group was significantly younger than the T2DM group, consistent with the natural history of these diseases. Since serum IGF-1 rises steeply during puberty, the older age distribution in T2DM would naturally elevate unadjusted IGF-1 levels relative to T1DM. This underscores the necessity of age adjustment when comparing IGF-1 between these groups.

Lower IGF-1 in two subtypes with divergent *β*-cell reserves is of mechanistic interest, but our data cannot identify the cause. In T1DM, low IGF-1 is primarily attributed to portal insulin deficiency. Subcutaneous insulin replacement does not restore the high portal-to-peripheral insulin gradient required for hepatocyte GH receptor expression, resulting in hepatic GH resistance and low IGF-1 output despite compensatory GH hypersecretion. This is one possible explanation for our T1DM findings, although GH was not measured; the weak association with residual C-peptide may reflect the restricted range of C-peptide values. In T2DM, however, endogenous insulin secretion is preserved. Under a simple portal-insulin model, one might expect normal or elevated IGF-1, as seen in some obesity studies. Yet, age- and sex-adjusted IGF-1 in T2DM was also lower than in the comparison group, in line with a report of reduced IGF-1 in adults with T2DM (14). This raises the possibility that portal insulin delivery *per se* is not the sole operative variable in T2DM and that mechanisms acting downstream of or parallel to insulin exposure contribute (15, 16); this remains a hypothesis.

We propose several non-exclusive hypotheses, none of which was tested here. First, selective hepatic insulin resistance may play a key role. Insulin resistance in T2DM is not uniform. While hyperinsulinemia reflects peripheral failure, hepatic insulin sensitivity, which governs GH-receptor expression, may be disproportionately impaired. The liver may fail to translate high portal insulin into GH responsiveness, mimicking the biochemical picture of insulin insufficiency seen in T1DM. Support for a selective hepatic GH lesion comes from liver-specific GH-receptor knockout mice, which develop low IGF-1, steatosis, and systemic insulin resistance (17). Second, chronic hyperglycemia may directly impair hepatic GH-receptor signaling. Both diabetes groups had a substantial glycemic burden (median HbA1c 7.9% in T1DM and 10.5% in T2DM), making glycemic exposure a plausible contributor. Third, inflammation and adipokines may contribute to this deficit. T2DM in this cohort had a numerically higher median BMI (22.1 kg/m^2^, vs 16.8 in T1DM and 17.4 in the comparison group; overall *P* = 0.133) and male predominance. Obesity-related inflammation and altered adipokine signaling can blunt hepatic GH sensitivity, as proinflammatory cytokines induce hepatocyte GH resistance (18), and elevations in specific interleukins have been linked to reduced IGF-1 in obese children (19).

Furthermore, changes in IGF-binding proteins (IGFBPs) may reduce IGF-1 bioavailability. In T1DM, elevated IGFBP-1 and IGFBP-2 lower bioactive IGF-1, and a similar profile has been reported in obese, insulin-resistant children, suggesting a comparable mechanism in T2DM. Additionally, hepatic steatosis cannot be excluded as a confounder. Non-alcoholic fatty liver disease is common in pediatric T2DM. While liver enzymes, including transaminases, were numerically higher in our T2DM group, differences were not significant (see Supplementary Material); however, subclinical steatosis may still influence IGF-1 output in the absence of direct imaging data. The dissociation between C-peptide and IGF-1 in our cohort further suggests that these markers capture distinct dimensions of metabolic health. Because C-peptide contributed to subtype classification, its difference between subtypes is expected by design, and whether C-peptide and IGF-1 carry complementary prognostic information cannot be judged from these cross-sectional data.

Variation in WFS1, which encodes wolframin, has been implicated in diabetes susceptibility and, in mouse models, in GH/IGF-1 signaling (20, 21); a genetic contribution is possible but speculative and was not examined here. Our supportive analysis using an external reference is consistent with lower IGF-1 in T1DM (median SDS −0.32, *P* = 0.009); in T2DM the SDS was numerically in the same direction but not significantly different from zero (*P* = 0.33, *n* = 13). The external reference was derived with a different assay and population, so these results are supportive only.

This study has several limitations, many stemming from its retrospective design. The T2DM group was small, reflecting the inherently low incidence of prepubertal T2DM and the later, puberty-associated onset typical of youth-onset T2DM, which further narrowed the pool of eligible patients at a single center. For the same reason, Tanner stage was not systematically recorded in clinical documentation, so chronological age was used as a proxy for pubertal variation. Diabetes duration and treatment regimens were not consistently recorded, and HbA1c reflected only a single time point rather than cumulative exposure. Liver function tests, including transaminases, did not differ significantly (see Supplementary Material), but direct measures of steatosis were unavailable. Age was modeled linearly, as sample size precluded testing non-linear terms. Although islet autoantibody testing was performed, methodological changes in reagents and instruments across the prolonged study period prevented the inclusion of autoantibody data in the analysis. T1DM classification therefore relied on clinical presentation, plasma glucose criteria, C-peptide levels, and treatment course. The study was a retrospective convenience sample without an *a priori* sample-size calculation, and only 14 children had T2DM; we therefore report confidence intervals to convey precision rather than *post hoc* power, and all T2DM and subgroup findings should be regarded as exploratory. Finally, the non-diabetic comparison group comprised children attending child health-care check-ups at a single hospital rather than a population-based sample, and glucose tolerance was not tested by OGTT, potentially introducing selection bias and misclassification of non-diabetic status, though this is partly mitigated by the external SDS reference.

## 5 Conclusions

After adjustment for age and sex, serum IGF-1 was lower in children with T1DM and in children with T2DM than in a non-diabetic comparison group. Whether the magnitude of the reduction differs between the two subtypes could not be determined because of the small T2DM sample. These exploratory findings are hypothesis-generating and warrant prospective studies that include pubertal staging, BMI-SDS, and measurements of the GH axis.

## Data Availability

The raw data supporting the conclusions of this article will be made available by the authors, without undue reservation.
