# Supplementary material for: Serum insulin-like growth factor-1 is lower in children with type 1 and type 2 diabetes mellitus than in a non-diabetic comparison group after adjustment for age and sex: a cross-sectional study
Source: Front Pediatr. 2026 Oct 13;14:1948533. doi: 10.3389/fped.2026.1948533 (PMC13710310; doi:10.3389/fped.2026.1948533)
Supplement: Supplementary file 1 [file Datasheet1.docx]

# Supplementary Material

Serum Insulin-like Growth Factor-1 Is Lower in Children With Type 1 and Type 2 Diabetes Mellitus Than in a Healthy Comparison Group After Adjustment for Age and Sex

Supplementary Table S1. IGF-1 SDS relative to an external pediatric reference population (Bidlingmaier et al. [15]).

| Metric | T1DM (n=57) | T2DM (n=14) | P |
| --- | --- | --- | --- |
| Median IGF-1 SDS [IQR] | −0.32 [−1.17, 0.40] | −0.54 [−1.14, 0.49] | 0.81ᵃ |
| Mean ± SD | −0.58 ± 1.52 | −0.36 ± 1.42 | — |
| SDS < 0, % | 68.4% | 49.9% | — |
| SDS < −1 (mild deficit), % | 31.6% | 28.6% | — |
| SDS < −2 (marked deficit), % | 14.0% | 14.3% | — |
| P vs reference population (SDS=0)ᵇ | 0.009 | 0.33 | — |

IGF-1 values were converted to age- and sex-specific SDS using the LMS reference of Bidlingmaier et al. [15] (N=15,014; IDS-iSYS chemiluminescence immunoassay). Only children with complete age and sex data were included in the analysis (T1DM, 57 of 70; T2DM, 14 of 14). ᵃ Mann-Whitney U test, T1DM vs T2DM SDS. ᵇ One-sample Wilcoxon signed-rank test against a reference-population median of 0. SDS, standard deviation score; IQR, interquartile range.
